# Large and Giant Unilamellar Vesicle(s) Obtained by Self-Assembly of Poly(dimethylsiloxane)-*b*-poly(ethylene oxide) Diblock Copolymers, Membrane Properties and Preliminary Investigation of Their Ability to Form Hybrid Polymer/Lipid Vesicles

**DOI:** 10.3390/polym11122013

**Published:** 2019-12-04

**Authors:** Martin Fauquignon, Emmanuel Ibarboure, Stéphane Carlotti, Annie Brûlet, Marc Schmutz, Jean-François Le Meins

**Affiliations:** 1 Université de Bordeaux, CNRS, Bordeaux INP, LCPO, UMR 5629, F-33600 Pessac, France; Martin.Fauquignon@enscbp.fr (M.F.); ibarbour@enscbp.fr (E.I.); carlotti@enscbp.fr (S.C.); 2 Laboratoire Léon Brillouin, UMR12 CEA-CNRS, CEA Saclay, F-91191 Gif-sur-Yvette CEDEX, France; annie.brulet@cea.fr; 3 Institut Charles Sadron, UPR 22 CNRS, Université de Strasbourg, 23 rue du Loess, 67034 Strasbourg, France; marc.schmutz@ics-cnrs.unistra.fr

**Keywords:** block copolymer, phospholipid, giant hybrid unilamellar vesicles, micropipette, self-assembly

## Abstract

In the emerging field of hybrid polymer/lipid vesicles, relatively few copolymers have been evaluated regarding their ability to form these structures and the resulting membrane properties have been scarcely studied. Here, we present the synthesis and self-assembly in solution of poly(dimethylsiloxane)-*block*-poly(ethylene oxide) diblock copolymers (PDMS-*b*-PEO). A library of different PDMS-*b*-PEO diblock copolymers was synthesized using ring-opening polymerization of hexamethylcyclotrisiloxane (D3) and further coupling with PEO chains via click chemistry. Self-assembly of the copolymers in water was studied using Dynamic Light Scattering (DLS), Static Light Scattering (SLS), Small Angle Neutron Scattering (SANS), and Cryo-Transmission Electron Microscopy (Cryo-TEM). Giant polymersomes obtained by electroformation present high toughness compared to those obtained from triblock copolymer in previous studies, for similar membrane thickness. Interestingly, these copolymers can be associated to phospholipids to form Giant Hybrid Unilamellar Vesicles (GHUV); preliminary investigations of their mechanical properties show that tough hybrid vesicles can be obtained.

## 1. Introduction

Hybrid polymer vesicles are emerging and promising systems that spark an increasing interest from different scientific communities (chemists, physico-chemists, biochemists, biophysicists, and pharmacists) due to their high potentiality in different application fields such as controlled and targeted drug delivery, biomolecular recognition within biosensors for diagnosis, functional membranes for artificial cells, and the development of bioinspired micro/nanoreactors [1,2]. Thus far, relatively little information is available about the structure, chemical nature, and molar mass of copolymers that could mix ideally with phospholipids. Most commonly, poly(butadiene) [3,4,5,6,7,8,9,10,11,12] and poly(dimethylsiloxane) [13,14,15,16,17,18,19,20] have been used as hydrophobic block, while studies with poly(isobutene) [21,22,23], poly(caprolactone) [24,25], poly(isoprene) [26], and poly(laurylacrylate) [27] have also been reported. Poly(ethylene oxide) is by far the most used hydrophilic block, although few studies report the use of hydrophilic thermo-responsive blocks such as poly(2-isopropyl-2-oxazoline) [28] and poly(ethylene glycoldiacrylate) [27]. In addition, diblock copolymers are almost exclusively used, except in a previous study of our group where grafted and triblock copolymers based on PDMS and PEO were considered [16,17,18,19].

Finally, despite an increasing number of studies, there is still a need of systematic approach to precisely decipher the molecular parameters necessary to associate in an efficient way (i.e., optimized or modulated membrane properties) copolymers and lipids. For instance, one of the main ideas is to obtain vesicular structure with lipid in the membrane but presenting high mechanical stability. This can be of great interest for instance in biomedical application such as drug delivery, in which vesicles used as cargos have to withstand stress in the blood stream.

Results available on mechanical properties of hybrid vesicles are scarce. It has been reported, in a system based on diblock poly(butadiene)-*b*-poly(ethylene oxide) and 1-palmitoyl-2-oleoyl-glycero-3-phosphocholine (POPC) (93/7 *w/w*), that membrane mechanical properties seem to be modulated between those of pure polymersomes and liposomes [29]. Vesicles composed of mixture of a PDMS-*g*-PEO grafted copolymer and 1,2-dipalmitoyl-sn-glycero-3-phosphocholine (DPPC) (57/43 *w/w*) present mechanical properties similar to pure PDMS-*g*-PEO vesicles. This could be interesting but the lipidic phases are in the gel state, which is not ideal in terms of biomimetic character and permeability. Moreover, this copolymer itself leads to polymersomes with very thin membrane close to those of liposomes and the gain in mechanical properties is weak [30].

In the only study describing the association of triblock copolymer and lipid in a fluid state (poly(ethylene oxide)-*b*-poly(dimethylsiloxane)-*b*-poly(ethylene oxide) with POPC), it has been reported that the resulting hybrid vesicles were even more fragile than pure liposomes, although membrane thickness of the polymersomes and lipid/polymer fraction were comparable to those of PBut-PEO/POPC. It has been surmised that mixture of hairpin and stretch conformations of polymer chains in the membrane could make obtaining stable edifices with lipids difficult [15].

As it is difficult to extract some tendencies from the literature regarding the association of copolymer and lipid and the resulting mechanical properties of hybrid membrane, we decided to extend the previous study made on triblock copolymers to diblock copolymer based on poly(dimethylsiloxane) and poly(ethylene oxide) as a systematic study. Since the diblock copolymers ensure a bilayer conformation of polymer chains in the membrane as it is for the lipids, the issues observed with hybrid vesicle formulated with triblock copolymers are not expected. Direct comparison of the architecture effect is then possible as the chemical nature of the copolymer is unchanged. A series of diblock copolymers PDMS-*b*-PEO was synthesized by ring-opening polymerization of D3, functionalization of PDMS chain, and further coupling with alkyne end-functionalized poly(ethylene oxide) using Huisgen’s coupling. We targeted different molar masses keeping hydrophilic weight fraction in the range of 25–35%, which has been empirically shown to favor the formation of polymersomes during self-assembly, for neutral coil–coil diblock copolymer [31]. The self-assembled structure in solution of copolymers synthesized was studied by different techniques: dynamic and static light scattering, cryo-transmission electron microscopy, and small angle neutron scattering. To access to the membrane mechanical properties, giant polymersomes were prepared, allowing the use of micropipette aspiration technique. It has to be noted that relatively few data are available regarding mechanical properties of polymersomes, such as area compressibility modulus, bending modulus, and lysis stress and strain, although scaling laws have been established with membrane thickness [32,33]. These parameters were thus measured and compared to those evaluated in a previous study on triblock copolymers. Finally, experiments on the ability of these diblock copolymers to form giant hybrid vesicles with POPC as well as preliminary study on the resulting mechanical properties were initiated. 

## 2. Materials and Methods 

### 2.1. Materials

Hexamethylcyclotrisiloxane (D3) was purchased from Acros (Illkirch France) and purified on CaH_2_ before use. α-hydroxy-PEO were obtained from Polysciences (2000 g·mol^−1^, Niles, MI, USA), Creative Pegworks (1000 g·mol^−1^, Durham, NC, USA), Sigma Aldrich (750 and 550 g·mol^−1^, Haverhill, MA, USA), and Alfa Aesar (350 g·mol^−1^, Haverhill, MA, USA) and used without purification. Chloro-(3-chloropropyl)dimethylsilane was purchased from ABCR (Karlsruhe, Germany) and purified by distillation on CaH_2_. Sodium azide (NaN_3_), copper bromide, sec- or n-butyllithium, and propargylbromide were purchased from Sigma Aldrich (Haverhill, MA, USA) and used without purification. THF, purchased from VWR (Radnor, PA, USA), was dried over Na/benzophenone and cryo-distilled just before use. Cyclohexane and chloroform were purchased from VWR and used without purification.

All copolymers were characterized by ^1^H NMR in CDCl_3_ and size exclusion chromatography in THF. Synthesized PDMS polymers were characterized by ^1^H NMR in CDCl_3_ and by SEC in toluene.

### 2.2. ω-chloro-PDMS Synthesis: Example of PDMS_27_-Cl

First, 7.5 g of hexamethylcyclotrisiloxane were dried over CaH_2_ during 24 h at 80 °C and then cryo-transferred into a preliminary tared bottom-round flask. Seven grams of monomer were collected (31.5 mmol, 1 equivalent (eq).) and 30 mL of freshly distilled THF were added. The polymerization was initiated by adding under argon flux 2.7 mL (3.5 mmol, 0.11 eq.) of a solution of butyllithium at 1.3 M in cyclohexane/hexane. The solution was stirred at room temperature for 2 h. Then, the reaction was quenched by adding 1.13 mL (6.93 mmol, 0.22 eq.) of chloro-(3-chloropropyl)dimethylsilane and stirred for 12 h. The product was recovered by solvent evaporation, dissolved again in cyclohexane and filtered over 0.22 µm PTFE filters to remove salts. After cyclohexane evaporation, the product was washed twice with a minimum of methanol. The product was finally dried under vacuum overnight. The polymer was obtained as a colorless oil (6.5 g, 3.3 mmol, yield: 93 wt.%) and was characterized by ^1^H NMR, SEC in toluene, and FT-IR (Vertex 70, from Bruker, Billerica, MA, USA). 

### 2.3. ω-azido-PDMS Synthesis: Example of PDMS_27_-N_3_

First, 5.5 g of PDMS_27_-Cl (2.8 mmol, 1 eq.) were suspended in 40 mL of a mix 50/50 vol. dimethoxyethane (DME) and dimethylformamide (DMF). One gram (15 mmol, 5.4 eq.) of NaN_3_ was added. The mixture was heated at 90 °C for 48 h. Once cooled down, the product was diluted in 100 mL of pentane and washed three times with 100 mL of water. The organic phase was collected, solvents were evaporated, and the product was dried under vacuum overnight. The final product was obtained as a colorless oil (5.2 g, 2.6 mmol, yield: 95 wt.%) and was characterized by ^1^H NMR, SEC in toluene, and FT-IR.

### 2.4. α-alkyne-PEO Synthesis: Example of PEO_17_-Alkyne

Three grams of commercial methoxy terminated PEO_17_ (4 mmol, 1 eq.) were dissolved in 30 mL of freshly distilled THF. Then, 0.2 g of NaH (8 mmol, 2 eq.) were added and the mixture was stirred at room temperature for 1 h. Next, 4.5 mL (40 mmol, 10 eq.) of a solution of propargyl bromide at 80 wt.% in toluene were added. The mixture was stirred at room temperature and allowed to react for 24 h. The mixture was concentrated, diluted again with 100 mL of dichloromethane, washed three times with 100 mL of water, and finally dried under vacuum overnight. The final product was obtained as a yellowish powder (2.8 g, 3.7 mmol, yield: 93 wt.%) and was characterized by ^1^H NMR and SEC in THF.

### 2.5. PDMS-b-PEO Synthesis: Example of PDMS_27_-b-PEO_17_

Five grams of PDMS_27_-N_3_ (2.3 mmol, 1 eq.) and 2.1 g of PEO_17_-Alkyne (2.8 mmol, 1.2 eq.) were dissolved in 100 mL of toluene in a round bottom flask. The mixture was subjected to two cycles of freezing/degassing. Then, 0.119 g (0.69 mmol, 0.3 eq.) of pentamethyldiethylenetriamine (PMDETA) and 0.1 g (0.69 mmol, 0.3 eq.) of CuBr were added. The mixture was submitted to two cycles of freezing/degassing. The reaction was started by heating at 80 °C for 24 h. The mixture was cooled down and filtered using 0.22 µm PTFE filters. The solvent was evaporated and the product was solubilized in dichloromethane. The purification was performed on silica column using a 7 vol.% mix of methanol with dichloromethane as eluent phase. The product was collected, the solvent was evaporated, and the product was dried under vacuum overnight. The final product was obtained as a viscous to gelled oil (5.5 g, 2.0 mmol, yield: 87 wt.%) and was characterized by ^1^H NMR and SEC in THF.

### 2.6. ^1^H Nuclear Magnetic Resonance Characterization

All ^1^H NMR spectrum were recorded at 298 K on a Avance 400 spectrometer from Bruker (Wissembourg, France) operating at 400 MHz using a D1 of 1 s and 32 scans. Products were dissolved in deuterated chloroform at 10 mg·mL^−1^_._

### 2.7. Size Exclusion Chromatography

Size exclusion chromatography (SEC) analyses were performed on an Ultimate 3000 system from Thermo Fischer Scientific (Waltham, USA) with a diode array detector (UV), equipped with a multi-angle light scattering (MALS) detector, a differential refractive index detector (dRI) from Wyatt Technology, and a three-column set of TOSOH TSK HXL gel (G2000, G3000 and G4000) with exclusion limits from 200 to 400,000 g·mol^−1^. PEO homopolymers and copolymers were solubilized and injected in THF. PDMS homopolymers were solubilized and injected in toluene. In both cases, the flow rate was set at 1 mL·min^−1^ and trichlorobenzene were used as flow marker. EasiVial kit of polystyrenes from Agilent was used as standard (162 to 364,000 g·mol^−1^) to determine the polymer’s number average molar mass ($\bar{M_{n}}$) and dispersity (Ð). Data were processed using Astra software (Wyatt technology France, Toulouse, France).

### 2.8. Process to Obtain Self-assembled Nano-structures

We used film rehydration process followed by extrusion through a polycarbonate membrane (21 times, 100 nm pore size). Briefly, copolymers were dissolved in chloroform. The solutions were then vacuum-dried until complete solvent evaporation and re-suspended in milli Q water, under gentle stirring. Afterwards, the extrusion process was performed. Concentration of the vesicular suspension was 1 mg·mL^−1^ for light scattering experiments and Cryo-TEM and 10 mg·mL^−1^ for SANS.

### 2.9. Dynamic and Static Light Scattering

Self-assembled nanostructures were characterized by dynamic light scattering (DLS) at 90° using a Zetasizer Nano ZS90, (Malvern, Orsay, France) and static light scattering (SLS) using an ALV/CG-3 multi-tau goniometer in a range of angles θ from 20° to 150°, full digital correlator in combination with a Spectra Physics laser (emitting vertically polarized light at λ = 632.8 nm). Temperature was controlled at 25 °C.

Measurements of refractive increment index were performed on differential refractometer from WYATT Technology (Optilab rEX and HELEOS-II).

### 2.10. Small Angle Neutron Scattering

The Small Angle Neutron Scattering (SANS) experiments were carried out at the PACE spectrometer at Laboratoire Léon Brillouin CEA, Saclay, France. SANS was performed on systems for which vesicular structure were established by DLS, SLS, and Cryo-TEM. Data analysis was done by fitting models to fit the scattering intensity curves with vesicle form factors, using the SasView program (http://www.sasview.org/).

### 2.11. Cryo Transmission Electron Microscopy

The vitrification of the samples was carried out in a homemade vitrification system. The chamber was held at 22 °C and the relative humidity at 80%. A 5 µL drop of the sample (1–2 mg·mL^−1^) was deposited onto a lacey carbon film covered grid ((Ted Pella, Redding, CA, USA) rendered hydrophilic using an ELMO glow discharge unit (Cordouan Technologies, Bordeaux, France). The grid was automatically blotted to form a thin film which was plunged in liquid ethane held at −190 °C by liquid nitrogen. That way, a vitrified film was obtained, in which the native structure of the vesicles was preserved. The grid was mounted onto a cryo holder (Gatan 626, Pleasanton, CA, USA) and observed under low dose conditions in a Tecnai G2 microscope (FEI, Eindhoven, Netherland) at 200 kV. Images were acquired using an Eagle slow scan CCD camera (FEI).

### 2.12. Giant Unilamellar Vesicle Preparation

All giant unilamellar vesicles were prepared using electroformation method reported by Angelova [34] at room temperature. Briefly, to obtain the solutions of polymers or the mixture of POPC and copolymers, the hybrid vesicles were prepared in chloroform at 1 mg·mL^−1^. Fifty microliters of the solution were spread gently on ITO glass plates, and dried under vacuum for 3 h to remove any trace of organic solvent. Then, the ITO glass plates were connected to an AC voltage and submerged in sucrose solution at 100 mM. For all systems used, a sinusoidal tension (2 V, 10 Hz) was applied during 75 min. For better visualization of the vesicles, or identification of the phases in the case of GHUVs, fluorescent probes were used: PDMS-nitrobenzoxadiazole (PDMS-NBD) (1 mol.%) for polymer phase (synthesis protocol is available in Scheme S1 and characterization in Figure S2) and 1,2-dioleoyl-sn-glycero-3-phosphoethanolamine-N-(lissamine rhodamine B sulfonyl) (DOPE-Rhod) at 0.1 mol.% for lipid phases.

### 2.13. Micropipette Experiments

The area compressibility modulus, lysis strain, and cohesive energy density of the GUV were determined by micropipette aspiration technique [35]. Micropipette were obtained by stretching borosilicate capillaries (1 mm OD, 0.58 mm ID) from WPI, using a pipette puller (Sutter Instrument P-97) and the desired diameter of the pulled pipettes were obtained using a micro-forge Narishige MF-900. Micropipettes were coated with bovine serum albumin (BSA) to prevent vesicle adhesion (BSA fraction V purchased from Aldrich). The vesicle tension was controlled using a homemade hydraulic watertight setup, and the micropipette was controlled using a micromanipulator (Eppendorf, Patchman NP2). The membrane tension was calculated classically from the Laplace equation (Equation (1))

(1) $$\sigma =\frac{\Delta P}{2}\frac{R_{P}}{1-\frac{R_{P}}{R_{V}}}$$

R_p_ and R_v_ are the micropipette and vesicle radius (outside the micropipette). ΔP is the suction pressure on the micropipette. The surface area strain (α) of the membrane is defined as:

(2) $$\alpha =\frac{A-A_{0}}{A_{0}}$$

where $A_{0}$ is the membrane area of the vesicle at the lower suction pressure α, which can be calculated from the increase in projection length ΔL of vesicle inside the capillary tip according to Equation (3) [35].

(3) $$\alpha =\frac{1}{2}{\left(\frac{R_{P}}{R_{V}}\right)}^{2}\left(1-\frac{R_{P}}{R_{V}}\right)\frac{\Delta L}{R_{P}}$$

The surface area strain can be linked to the membrane tension through two contributions: one at low tension where smoothing of thermal bending undulations dominates the apparent expansion (first term of Equation (4)), and one at high tension, where membrane undulations are completely suppressed and membrane area increases as the result of increased spacing between molecule (second term of Equation (4)), giving access to area compressibility modulus K_a_.

(4) $$\alpha =\frac{k\;T}{8{\;\pi \;K}_{b}}\ln\left(1+\frac{A_{0}\;\sigma }{24{\;\pi \;K}_{b}}\right)+\frac{\sigma }{K_{a}}$$

A very detailed procedure was published in Journal Of Visualized Experiments (JoVE by our group (https://www.jove.com/video/60199/obtention-giant-unilamellar-hybrid-vesicles-electroformation).

## 3. Results and Discussion

### 3.1. Block Copolymer Synthesis and Characterization 

**Scheme 1:**
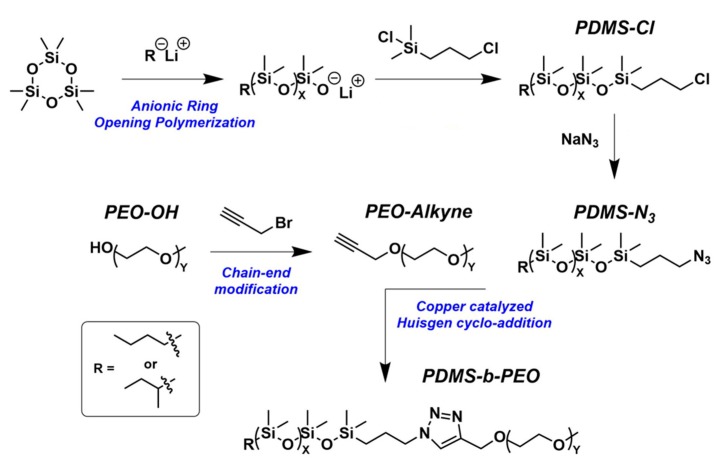
General synthesis procedure to obtain PDMS-*b*-PEO copolymers.

The general methodology of the synthesis is illustrated in Scheme 1. ω-Chloro-PDMS is synthesized by anionic ring-opening polymerization of hexamethylcyclotrisiloxane monomer (D3), in anhydrous THF at 80 °C. We used either sec- or n-butyllithium as initiator, dissolved in cyclohexane, which gave similar results in term of control of molar mass and dispersity. The chain end functionalization was obtained using chloro-(3-chloropropyl) dimethylsilane as terminating agent during one night at −80 °C. The PDMS-Cl was then purified, collected, and analyzed by ^1^H NMR and SEC in toluene. Results are shown in Figure S1A,B. The chloro group was then converted into azido group by nucleophilic substitution using sodium azide, to finally obtain ω-azido-PDMS. The azidation was checked by Infrared spectroscopy; an example of spectrum is indicated in Figure S3. 

To obtain α-alkyne-PEO, hydroxyl groups of PEO monomethyl ether were allowed to react with propargyl bromide. The products were purified and washed. The NMR spectra of the different α-alkyne-PEO obtained are illustrated in Figure S4. The NMR spectra of the different α-hydroxy-PEO as well as the SEC chromatograms are illustrated in Figure S5. An example of chromatogram of α-alkyne-PEO compared to precursor α-hydroxy-PEO is also illustrated in Figure S4B.

The block copolymers were obtained by coupling ω-azido-PDMS and α-alkyne-PEO using Copper-Catalyzed Azide-Alkyne Cycloaddition (CuAAC). The copolymers obtained were characterized by ^1^H NMR in CDCl_3_ and size exclusion chromatography (Figure S6) a good agreement between the two techniques was obtained regarding the molar mass of the copolymers. The disappearance of the azido function was checked by IR spectroscopy attesting to the efficiency of the coupling reaction (Figure S3). Different molar masses and hydrophilic fractions were targeted, the aim being to obtain a series of copolymers able to form a vesicular structure in aqueous solution and determine the parameters that govern such self-assembly. A list of copolymers synthesized is available in Table S1. We report only in the main text the characteristic of vesicles forming block copolymers (Table 1).

In the literature, studies reporting on the synthesis and self-assembly of PDMS-*b*-PEO copolymers into bilayer and vesicular structures are relatively scarce, despite the simplicity of the architecture and conformation of the chains, i.e., coil-coil diblock. Very short PDMS-*b*-PEO copolymers, with hydrophilic weight fraction (f) of 40–60% have been reported to form vesicular structures [36]. In another study, very short PDMS_8_-*b*-PEG_6_ (f = 30%) led to vesicles using nanoprecipitation method (copolymer dissolved in THF and progressive addition of water). However, the obtained objects were unstable over time, whereas stable vesicles, according to the authors, were obtained with the same process using PDMS_8_-*b*-PEG_23_ (f = 63%) and PDMS_81_-*b*-PEG_23_ (f = 14%) [37]. It seems difficult from these experimental results to conclude on the molecular parameters necessary to obtain vesicular structures at equilibrium for such copolymers. 

Only the copolymers indicated in Table 1 led to the formation of or giant unilamellar vesicles by electroformation. A complete characterization of the nanostructure obtained by the extrusion hydration process was therefore performed. For all others copolymers, we were not able to obtain giant unilamellar vesicles by electroformation and therefore we did not focus on the characterization of the nanostructure obtained by hydration extrusion process. We were able to obtain vesicular structure for PDMS-*b*-PEO block copolymer with a molar mass of the PDMS block ranging 1000–2700 g·mol^−1^, and a hydrophilic weight fraction f around 30%.

The nanostructures were characterized by DLS, SLS, SANS, and Cryo-TEM. We focus on the PDMS-*b*-PEO copolymers able to form giant vesicles by electroformation: Si_23_EO_13_, Si_27_EO_17_, and Si_36_EO_23_. The copolymer Si_14_EO_8_ was not completely characterized since, as shown below, it formed polymersomes with poorly defined membranes.

**Figure 1:**
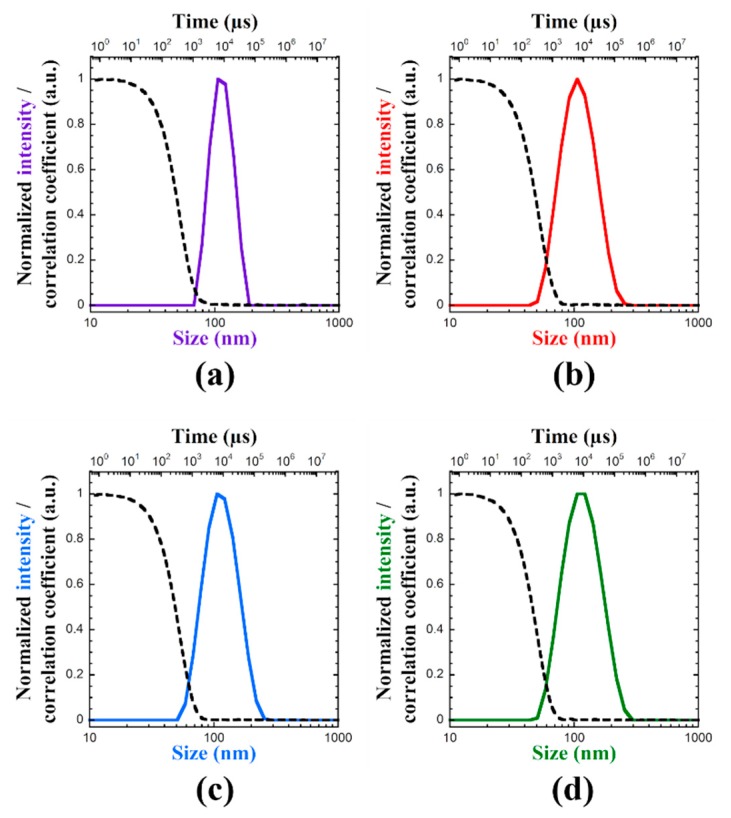
DLS at 90° with size distribution by intensity (plain lines) and autocorrelation function (dashed lines) of polymersomes made of: Si_14_EO_8_ (**a**); Si_23_EO_13_ (**b**); Si_27_EO_17_ (**c**); and Si_36_EO_23_ (**d**).

**Figure 2:**
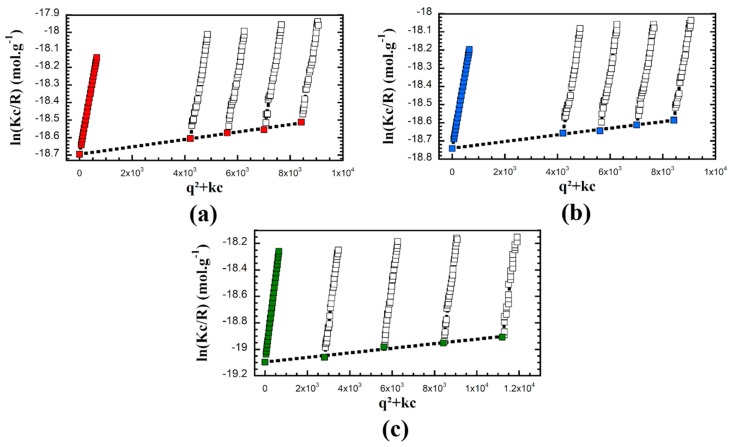
Relaxation frequency versus q^2^ for Si_23_EO_13_ (red squares), Si_27_EO_17_ (blue squares), and Si_36_EO_23_ (green squares).

The results obtained by DLS for the different copolymers, following the hydration extrusion process are illustrated in Figure 1a–d and Figure 2 and Table 2. All systems present a hydrodynamic diameter close to the pore size of the polycarbonate filter, which is classically observed for vesicular suspensions obtained from rehydration/extrusion process. The vesicles present a relatively narrow size distribution: the data could be treated using the second-order cumulant analysis. The polydispersity index (PDI) is defined as the ratio of the second-order cumulant coefficient to the square of the first one, PDI = µ2/<Γ>^2^. Values are reported in Table 2: the size distributions of vesicles are narrow.

**Figure 3:**
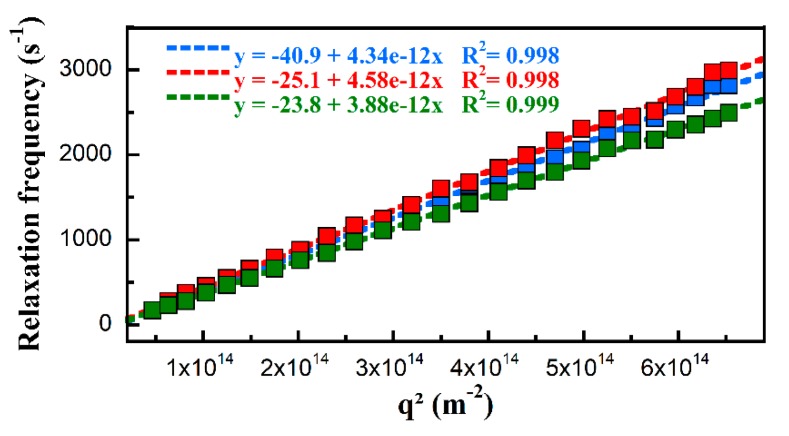
Multi-angle and multi-concentration Guinier plot showing intensity measured (white squares) and extrapolation to zero concentration and zero angle (colored squares) for polymersomes made from: Si_23_EO_13_ (**a**); Si_27_EO_17_ (**b**); and Si_36_EO_23_ (**c**).

The molar mass of the vesicles was determined by a complete study at multiple angles and concentrations (from 0.2 to 1 mg·mL^−1^) using static light scattering (Figure 3a–c). This allows knowing the dn/dc value of the vesicular suspensions, which was measured at various concentrations from 0.1 to 1 mg·mL^−1^. Measurements were replicated two or three times for repeatability. Radius of gyration (R_g_) was determined from Guinier plot. 

Hydrodynamic radius (R_H_) was estimated using the Stoke–Einstein relation (Equation (5)), where D_0_ is the diffusion coefficient determined from the slope of the q^2^ dependence of relaxation rate (<Γ> = D_0_q^2^).

(5) $$R_{H}=\frac{k_{B}\;T}{6{\;\pi \;\eta }_{s}{\;D}_{0}}$$

where k_B_ is the Boltzmann constant and η_s_ the solvent viscosity. The ratio R_g_/R_h_ is close to 1, as expected for vesicular structures. 

**Figure 4:**
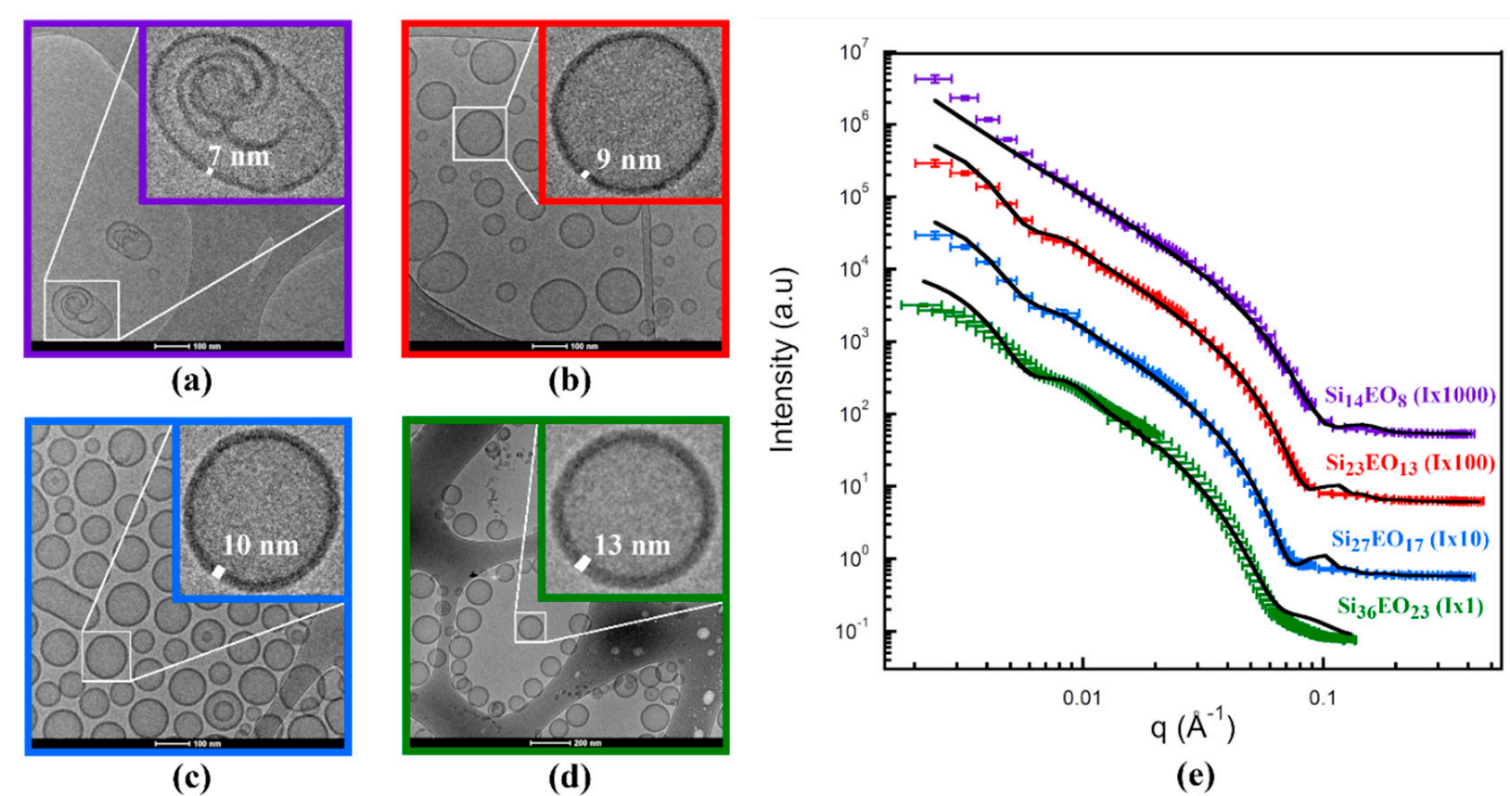
(**a**–**d**) Cryo-TEM images of polymersomes obtained for the different copolymers: (**a**) polymersomes obtained from Si_14_EO_8_; (**b**) polymersomes obtained from Si_23_EO_13_; (**c**) polymersomes obtained from Si_27_EO_17_; and (**d**) polymersomes obtained from Si_36_EO_23_. Inserts show zooms on single polymersomes with the measured membrane thickness. (**e**) SANS curves of the four diblocks. Black lines represent the best fits using the polydisperse vesicle model.

Cryo-TEM images of the four different copolymers are illustrated in Figure 4a–d. For Si_36_EO_23_, Si_27_EO_17_, and Si_23_EO_13_, rounded shaped vesicles were clearly observed. Membrane thickness could be estimated from an average of a few tens of vesicles. Values are reported in Table 2. In the case of Si_14_EO_8_, although the system could form a membrane-like structure, the global shape of the object was not well defined (see Figure 4a), and fewer objects were obtained than for the other copolymers on grids. Actually, the suspensions obtained from this copolymer were not stable with time, probably due to a too short PEO chain length, limiting the steric stabilization of the nanostructure. All the SANS curves of suspensions display the characteristic q^−2^ scaling law over a wide intermediate q scattering vector range (Figure 4e). All the curves are well fitted with a polydisperse vesicle form factor. The best fitting parameters are reported in Table S2. A fixed dispersity on the vesicle radius of 0.25 was used and gave good results. The membrane thickness (and its distribution) could be determined accurately for the four copolymers. 

**Figure 5:**
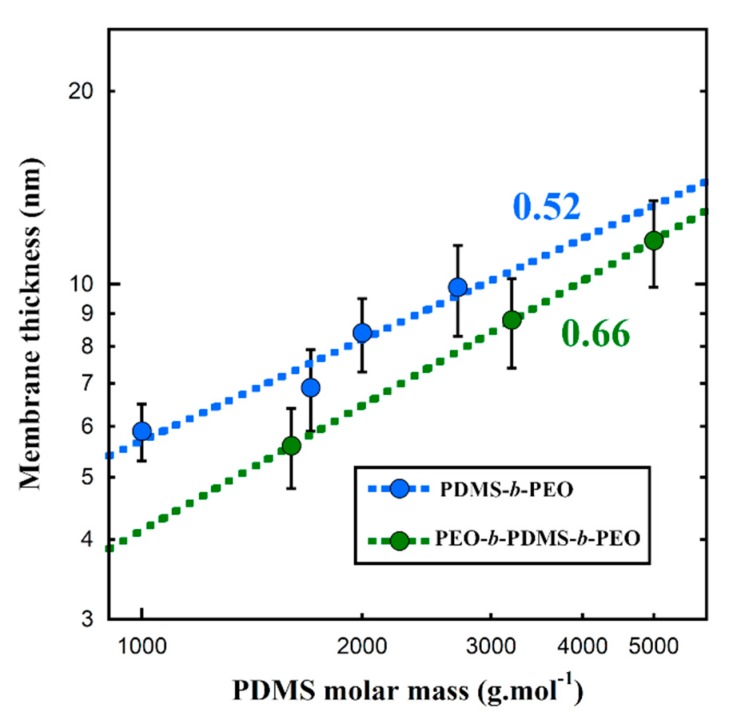
Membrane thickness versus PDMS molar mass plot. Membrane thickness was extracted from the SANS fits. It obeys a power law with an exponent value close to 0.5. Error bars represent the membrane thickness (Log-normal) distribution.

The membrane thickness varies from 5.9 to 9.9 nm and scales with molar mass as M^0.52^, as illustrated in Figure 5. This suggests that the PDMS chains are in a non-perturbed state in the membrane, as observed for poly(butadiene)-*b*-poly(ethylene oxide) [38] and poly(ethylethylene)-*b*-poly(ethylene oxide) [39]. This result also shows that the conformation of the chain in the membrane for diblock copolymers PDMS-*b*-PEO is different from those of triblock PEO-*b*-PDMS-*b*-PEO, where an exponent of 0.66 was reported, suggesting strong segregation regime [17] as also reported with PMOXA-*b*-PDMS-*b*-PMOXA triblock copolymers [40].

**Figure 6:**
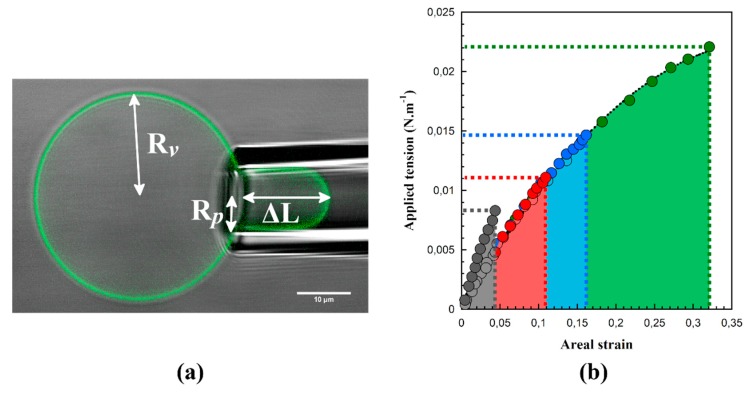
(**a**) LSCM (laser scanning confocal microscopy) image of GUVs under tension exerted by the micropipette. Intensity in the fluorescence green channel from PDMS-NBD and transmission channel were merged. R_v_, R_p_, and ΔL indicate vesicle radius, pipette radius, and variation of the tongue projection length, respectively. (**b**) Tension versus areal strain plot showing the typical curves obtained for GUVs composed of: POPC (grey dots), Si_23_EO_13_ (red dots), Si_27_EO_17_ (blue dots), and Si_36_EO_23_ (green dots). Fit of the data are represented in dotted black lines. The dotted colored lines indicate the lysis strain in the x-axis and the lysis stress in the y-axis.

The mechanical properties of the membrane formed by these diblock copolymers were evaluated by micropipette aspiration technique on giant vesicles. Typical curves obtained are shown in Figure 6. From these curves, we can determine the lysis strain and the lysis stress (see caption of Figure 6). 

Interestingly, the membrane resulting from the self-assembly of these diblock copolymers presents higher lysis strain than those obtained from triblock copolymer of similar membrane thickness [15], and slightly higher stretching modulus. Furthermore, beyond an area strain of 10%, the membrane tension evolves nonlinearly with area strain, as has already been observed for polymersomes obtained with PBut-*b*-PEO copolymers but with high molar mass of hydrophobic block (>7000 g·mol^−1^) [38]. However, although hysteresis (nonlinear elasticity effect) during cycle of the membrane tension has been observed for such systems, this was not the case here for the giant vesicles formed. Indeed, perfect reversibility was observed and stress–strain curves are superimposed during a cycle increasing/decreasing of the suction pressure. To our knowledge, this is the first time that such deformation (>30%) can be reached without hysteresis effect. The methodology to obtain area compressibility modulus, considering the nonlinearity observed, is explained in Appendix A.

The values obtained are reported in Table 3. Area compressibility moduli range from 113 to 121 mN·m^−1^. They are slightly higher than those obtained for membrane of triblock copolymers PEO-*b*-PDMS-*b*-PEO (80–90 mN·m^−1^) [15] and grafted copolymer PDMS-*g*-PEO (~95 mN·m^−1^) [30,41]. In the literature, it is commonly considered that the amplitude of the stretching modulus is linked to the interfacial tension [31,42] (i.e., dictated by chemical nature of the monomers and solvent alone) and independent of molar masses. Here, despite identical dimethylsiloxane monomers, a slight but non-negligible difference of K_a_ was observed between membranes formed from diblock and triblock copolymers. This suggests that the conformation of the chains in the membrane, and, consequently, the way they interact with surrounding water, has an importance on the amplitude of K_a_. 

**Figure 7:**
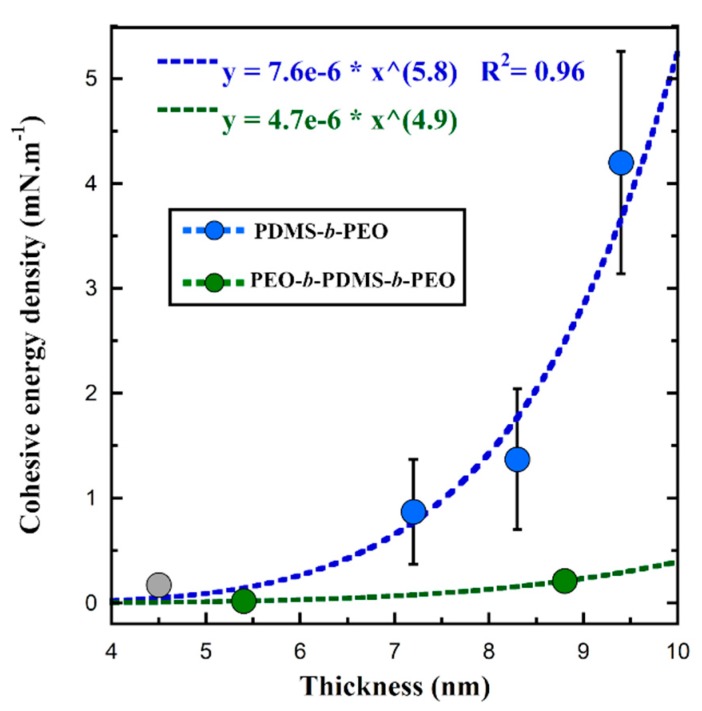
Cohesive energy density versus membrane thickness for GUV obtained from diblock PDMS-*b*-PEO copolymers. The grey circle illustrates a typical value of liposomes.

The amplitude of the area compressibility modulus and the lysis strain and stress of these GUV lead to high values of cohesive energy density (from 0.87 to 4.4 mN·m^−1^), and thus high toughness, far beyond those obtained with POPC liposomes (0.17 mN·m^−1^). The critical lysis strains measured are large (12–32%), and almost follow a power law with membrane thickness with an exponent of 3.6 (α_c_ ~ d^3.6^) (Figure S7). As membrane thickness d scaled with molar mass as d ~ M^0.5^, this implies that lysis strain scales as M^1.8^. This exponent is radically different from the scaling α_c_ ~ M^0.6^ obtained on a series of diblock copolymer PBut-*b*-PEO [38] and is hard to interpret. The toughness of the GUV seems to evolve with membrane thickness as a power law ~d^5.8^ (Figure 7). This make these diblock copolymers far more interesting than triblock copolymers PEO-*b*-PDMS-*b*-PEO, which displays relatively low lysis strain (<8% for membrane thickness around 8 nm) [15,43] for the development of hybrid polymer/lipid vesicles.

### 3.2. Giant Hybrid Unilamellar Vesicles Formation and their Mechanical Properties

Regarding the promising mechanical properties of such diblock copolymers, we performed a preliminary study aimed at evaluating their ability to incorporate lipids in the membrane, and the resulting mechanical properties by micropipette aspiration. We chose the diblock Si_27_EO_17_ as its membrane thickness (8.4 nm) is very close to the one formed by triblock copolymer investigated in previous studies (8.8 nm) [17]. 

**Figure 8:**
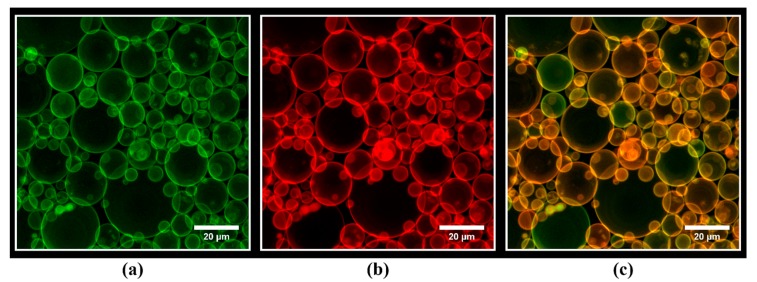
Confocal images obtained from GHUVs made of Si_27_EO_17_ mixed with 10 wt.% POPC. Images were obtained from a z-stack of different focal plans: (**a**) green channel from PDMS-NBD; (**b**) red channel from PE-Rhodamine; and (**c**) merged channels.

In the case of diblock Si_27_EO_17_, homogeneous hybrid vesicles at the microscale could be obtained by electroformation, as illustrated in Figure 8, with 10% *w/w* of POPC (which corresponds to ~26% mol). Similar morphologies have been obtained with the triblock copolymer [16].

**Figure 9:**
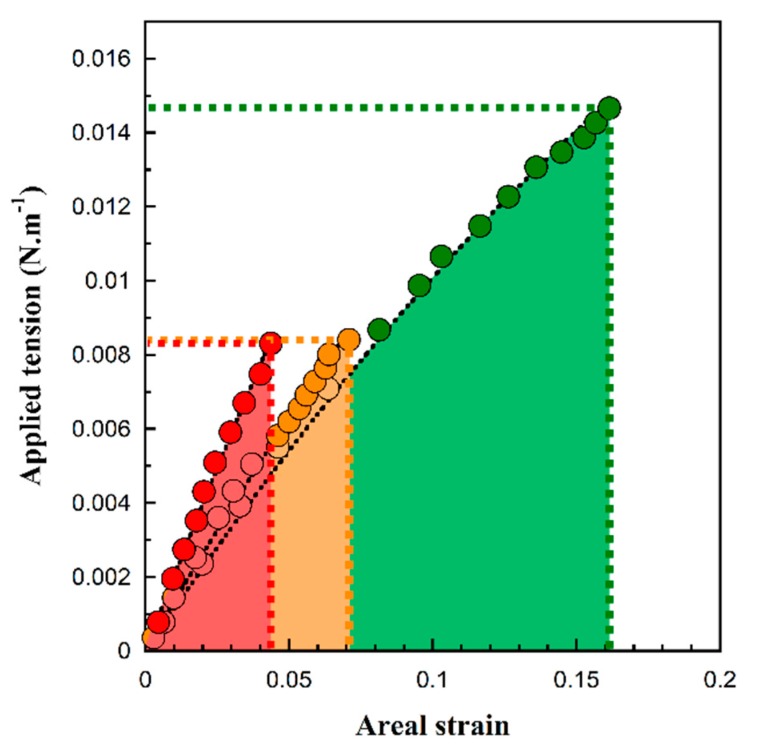
Typical micropipette aspiration curves obtained for GHUVs composed of Si_27_EO_17_ with 10% *w/w* POPC (orange circles). Properties of pure polymersomes (Si_27_EO_17_, green circles) and pure liposomes (POPC, red circles) are displayed for comparison. Dotted lines indicate the lysis strain in the x-axis and the lysis stress in the y-axis.

The mechanical properties of these GHUVs were evaluated by micropipette aspiration and compared to properties of pure liposome and polymersome membranes. Typical curves of membrane tension versus areal strain are illustrated in Figure 9. Area compressibility modulus (K_a_), Lysis strain and stress (α_L_ and σ_L_), and cohesive energy density (E_c_) were evaluated. Their values are reported in Table 4.

Interestingly, GHUVs of Si_27_EO_17_ with 10 wt.% POPC present intermediate mechanical properties between those of pure liposomes and polymersomes. Although the area compressibility modulus is not significantly modified, the lysis strain of hybrid vesicle and cohesive energy density are twice those of pure liposomes. In the case of GHUVs of triblock copolymer PEO-*b*-PDMS-*b*-PEO, extremely weak mechanical properties, even worse than those of pure liposomes, were obtained [15]. The origin of this unexpected phenomenon is not yet explained and may be in relation with the mixture of hairpin and extended chain conformation present in a membrane resulting from the self-assembly of triblock copolymer [15,40]. Here, the results obtained show that a bilayer conformation of the block copolymer is mandatory to really take advantage of the toughness of the polymersome membrane. 

## 4. Conclusions

In this work, a series of PDMS-b-PEO diblock copolymers of various molar masses and hydrophilic weight fraction was synthesized and their self-assembly was studied in aqueous media. Part of the synthesized copolymers, presenting a hydrophilic fraction around 30%, self-assemble into vesicular structures, whose membrane thickness is modulated via the molar mass of the hydrophobic block according to a scaling law d ~ M^0.5^, suggesting that PDMS chains are in a coil state (weak segregation regime) in the membrane. Interestingly, the membrane formed by this copolymer shows outstanding mechanical properties with high toughness and stretching elasticity far more pronounced than other polymersomes in the literature, for similar molar masses. Moreover, the mixing of the copolymer and phospholipid is efficient and leads to the formation of GHUVs, which present mechanical properties largely improved compared to the ones of liposome membrane, showing that these copolymers are excellent candidates to formulate hybrid vesicles. A complete study of the membrane physical properties such as fluidity, flexibility, and toughness, with copolymers of different molar masses and different lipid compositions is planned in a future work. Finally, this study shows that bilayer conformation of the copolymer chain in the membrane is one of the mandatory parameters to insure good mixing with the lipid and obtain hybrid structures with real mechanical benefits.

## Figures and Tables

**Table 1:** Molecular characterizations (SEC and NMR) of PDMS-*b*-PEO copolymers able to form vesicles.

		Si_36_EO_23_	Si_27_EO_17_	Si_23_EO_13_	Si_14_EO_8_
		PDMS_36_-*b*-PEO_23_	PDMS_27_-*b*-PEO_17_	PDMS_23_-*b*-PEO_13_	PDMS_14_-*b*-PEO_8_
**^1^H NMR**	$\bar{M_{n}}$ PDMS (g·mol^−^^1^)	2700	2000	1700	1000
	$\bar{M_{n}}$ PEO (g·mol^−^^1^)	1000	700	600	400
	$\bar{M_{n}}$ copolymer (g·mol^−^^1^)	4000	2900	2500	1600
	Hydrophilic weight fraction (%)	27	26	26	29
**SEC**	$\bar{M_{n}}$ PDMS (g·mol^−^^1^)	2700	2000	1700	1000
	Ð PDMS	1.09	1.18	1.26	1.12
	$\bar{M_{n}}$ PEO (g·mol^−^^1^)	1300	900	600	400
	Ð PEO	1.06	1.04	1.11	1.09
	Mn Copolymer (g·mol^−^^1^)	5000	3100	2500	1900
	Ð copolymer	1.04	1.11	1.15	1.13
	Hydrophilic weight fraction (%)	33	31	26	29

**Table 2:** Self-assembly parameters obtained by Multi-Angle Light Scattering (MALS) DLS/SLS, SANS, and cryo-TEM.

			Si_36_EO_23_	Si_27_EO_17_	Si_23_EO_13_	Si_14_EO_8_
**dn/dc**	g·mL^−^^1^		0.094	0.100	0.100	-
$\bar{M_{w}}$ **(c)**	g·mol^−^^1^	Guinier plot	2.0 × 10^8^	1.4 × 10^8^	1.3 × 10^8^	-
**R_g_**	nm		62	50	50	-
**N_agg_**	-		49,150	45,900	52,600	-
**R_h_**	nm	MALS	62	56	53	-
**PDI**	-		0.069	0.060	0.057	-
**R_g_/R_h_**	-		0.98	0.90	0.95	-
**Membrane thickness ± Standard deviation**	nm	Cryo-TEM	13.1 ± 1.5	10.0 ± 1.0	8.6 ± 0.9	7.1 ± 1.0
		SANS Fit	9.9 ± 1.6	8.4 ± 1.1	6.9 ± 1.0	5.9 ± 0.6

**Table 3:** Mechanical properties of GUV obtained for different PDMS-*b*-PEO GUVs, determined using micropipette aspiration technique.

	Si_36_EO_23_	Si_27_EO_17_	Si_23_EO_13_	POPC
**Membrane thickness d (nm) (from SANS)**	9.9 ± 1.6	8.4 ± 1.1	6.9 ± 1.0	4.5 ± 1.1
**Stretching modulus** **K_a_ (mN·m^−1^)**	113 ± 3	121 ± 8	118 ± 10	204 ± 13
**Lysis strain** **(%)**	32 ± 5	16 ± 4	12 ± 4	4 ± 1
**Lysis stress** **(mN·m^−1^)**	22 ± 2	15 ± 3	12 ± 3	8 ± 2
**Cohesive energy density (mN·m^−1^)**	4.20 ± 1.06	1.37 ± 0.67	0.87 ± 0.5	0.17 ± 0.09

**Table 4:** Mechanical properties determined using micropipette aspiration technique.

	Si_27_EO_17_	Si_27_EO_17_ + 10 wt.% POPC	POPC
**Stretching modulus** **K_a_ (mN·m^−1^)**	121 ± 8	125 ± 12	204 ± 13
**Lysis strain** **(%)**	16 ± 4	7 ± 2	4 ± 1
**Lysis stress** **(mN·m^−1^)**	15 ± 3	8 ± 2	8 ± 2
**Cohesive energy density** **(mN·m^−1^)**	1.37 ± 0.67	0.32 ± 0.15	0.17 ± 0.09

## Supplementary Materials

The following are available online at https://www.mdpi.com/2073-4360/11/12/2013/s1. Scheme S1: Synthesis scheme of the NBD-PDMS; Figure S1: (a,c) ^1^H NMR spectra of the different ω-chloro-PDMS synthetized in this study. (b) SEC chromatograms of ω-chloro-PDMS; Figure S2: (A) ^1^H NMR spectrum of PDMS-NBD. (B) SEC chromatograms of PDMS-NBD (red: RI detection; green: UV detection at 450 nm); Figure S3: (A) Comparison of IR spectra of ω-chloro-PDMS_36_, ω-azido-PDMS_36_, and diblock copolymer PDMS_36_-*b*-PEO_23_. (B) Zoom on characteristic peak of azide function at 2100 cm^−1^; Figure S4: (a,c) ^1^H NMR spectra of the different commercial ω-hydroxy-PEO used. (b) SEC chromatograms of ω-hydroxy-PEO; Figure S5: (a,c) ^1^H NMR spectra of the different ω-alkyne-PEO synthesized. (b) SEC of ω-alkyne-PEO_17_ and its precursor ω-hydroxy-PEO_17_; Figure S6: (a,c) ^1^H NMR spectra of the different PDMS-*b*-PEO synthesized. (b) SEC chromatograms of PDMS-*b*-PEO diblock copolymers; Figure S7: Lysis Strain versus membrane thickness for GUV obtained from PDMS-*b*-PEO diblock copolymers; Table S1: Molecular characteristics of the different copolymers PDMS-*b*-PEO synthesized in this study; Table S2: Fitting parameters of the SANS curves of block copolymers with vesicle form factor model.

## Appendix A

To obtain the area compressibility modulus, we take into account the nonlinearity of membrane tension σ in the stretching regime. We consider a membrane of area A_0_ and Length L_0_, and a stretched membrane of area A and length L, with linear strain ε defined as:

(A1) $$\varepsilon =\frac{L-{\;L}_{0}}{L_{0}}$$

The tension in the membrane is expressed as:

(A2) $$\sigma =\frac{E\;d}{1-v}\varepsilon$$

where E is the Young’s modulus of the membrane, d is the membrane thickness, and ν is the Poisson coefficient (which is 0.5 assuming incompressible material). Therefore, the tension can be written:

(A3) σ=2 E d ε

Assuming that the Young’s modulus is constant during deformation (which seems to be reasonable as no hysteresis, typical of nonlinear elasticity, was observed) and assuming volume conservation of the membrane, the following can be written:

(A4) $$d\;A={d\;A}_{0}=\mathrm{cst}.$$

Then,

(A5) $$d=d_{0}\frac{A_{0}}{A}\approx d_{0}\left(1-\alpha \right)$$

Therefore,

(A6) $$\alpha =\frac{\Delta A}{A_{0}}=\frac{L_{0}^{2}{\left(1+\varepsilon \right)}^{2}-L_{0}^{2}}{L_{0}^{2}}=2\varepsilon +\varepsilon^{2}$$

(A7) $$2\varepsilon +\varepsilon^{2}-\alpha =0$$

This leads to:

(A8) $$\varepsilon =\frac{1}{2}\alpha \left(1-\frac{1}{4}\;\alpha \right)$$

Finally, from Equations (A3) and (A8), the following can be written:

(A9) $$\sigma ={E\;d}_{0}\;\alpha \;\left(1-\alpha \right)\left(1-\frac{1}{4}\;\alpha \right)$$

Neglecting the term α^3^, this leads to:

(A10) $$\sigma =\mathrm{Ka}\;\alpha \left(1-\frac{5}{4}\alpha \right)$$

An analogous way to fit the data was used by Bermudez et al. [38] for a series of poly(butadiene)-*b*-poly(ethylene oxide) block copolymers where a nonlinear response of the membrane tension with α as well as a hysteresis phenomenon have been reported.

The data were fitted with this equation in the high-tension regime to obtain the area compressibility modulus (stretching modulus) K_a_.
