# Supplementary material for: Large and Giant Unilamellar Vesicle(s) Obtained by Self-Assembly of Poly(dimethylsiloxane)-b-poly(ethylene oxide) Diblock Copolymers, Membrane Properties and Preliminary Investigation of Their Ability to Form Hybrid Polymer/Lipid Vesicles
Source: Polymers (Basel). 2019 Dec 4;11(12):2013. doi: 10.3390/polym11122013 (PMC6960648; doi:10.3390/polym11122013)
Supplement: Supplementary file 1 [file polymers-11-02013-s001.pdf]

Supplementary informations for

Large and Giant Unilamellar vesicles obtained by self-assembly of poly(dimethylsiloxane)-*b*-poly(ethylene oxide) diblock copolymers, membrane properties and preliminary investigation of their ability to form Hybrid Polymer Lipid vesicles.

Martin Fauquignon[1], Emmanuel Ibarboure[1], Stéphane Carlotti[1], Annie Brûlet[2], Marc Schmutz[3] and Jean-François Le Meins[1,*]

[1] Université de Bordeaux, CNRS, Bordeaux INP, LCPO, UMR 5629, F-33600, Pessac, France
[2] Laboratoire Léon Brillouin, UMR12 CEA-CNRS, CEA Saclay, F-91191 Gif-sur-Yvette Cedex,
[3] Institut Charles Sadron, UPR 22 CNRS, Université de Strasbourg, 23 rue du Loess, 67034 Strasbourg, France
* Correspondence: lemeins@enscbp.fr; Tel.: (33)556846194

1. Synthesis protocol of PDMS-NBD

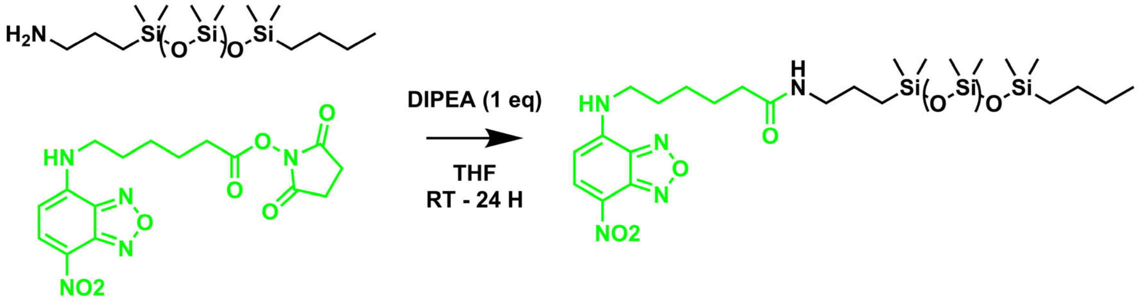

Scheme S1 :Synthesis scheme of the NBD-PDMS.

1 eq. of α-amino-PDMS purchased from Gelest was dissolved in THF and 1.2 eq. of N-hydroxysuccinimide ester-nitrobenzoxadiazole (NHS-NBD) was added. The coupling reaction was carried out with the presence of DIPEA during 24h at room temperature. The obtained products were then purified by dialysis (MWCO 2000 Da) against THF in order to remove probe in excess and base.

2. ¹H NMR and SEC characterization

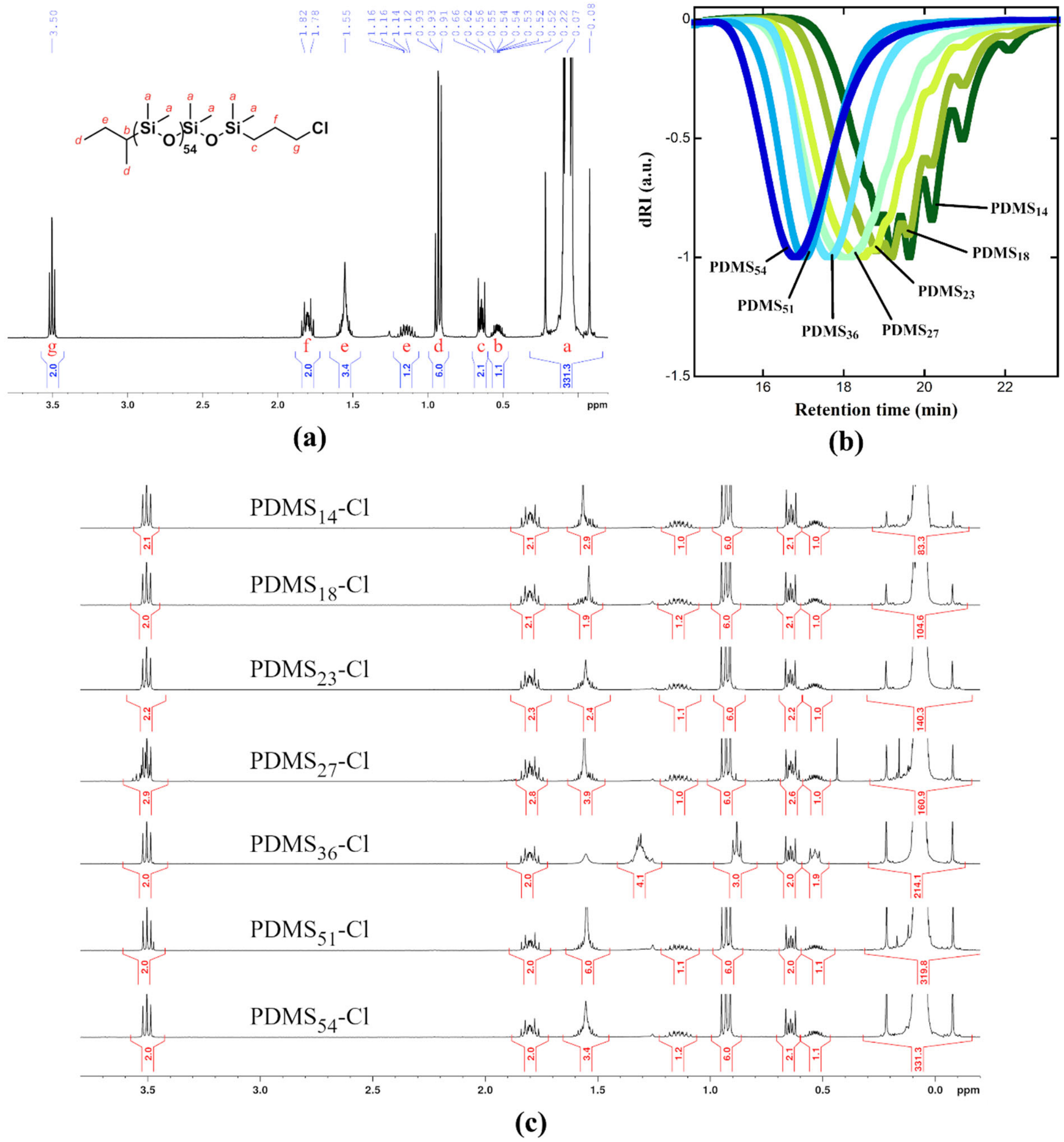

Figure S1. (a) and (c) ^{1}H NMR spectra of the different *ω*-chloro-PDMS synthetized in this study. (b) SEC chromatograms of *ω*-chloro-PDMS.

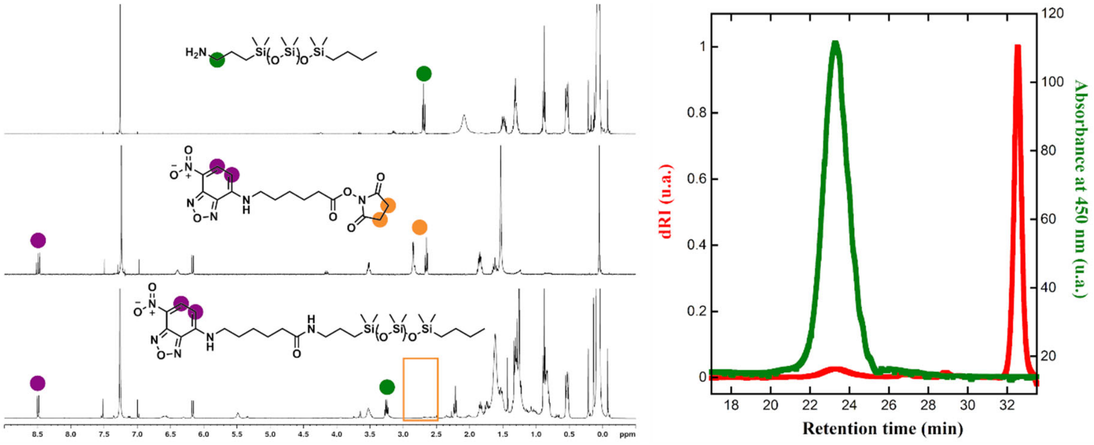

(a) **(b)**

Figure S2. A- ^{1}H NMR spectrum of PDMS-NBD. B- SEC chromatograms of PDMS –NBD (red : RI detection, green : UV detection at 450nm).

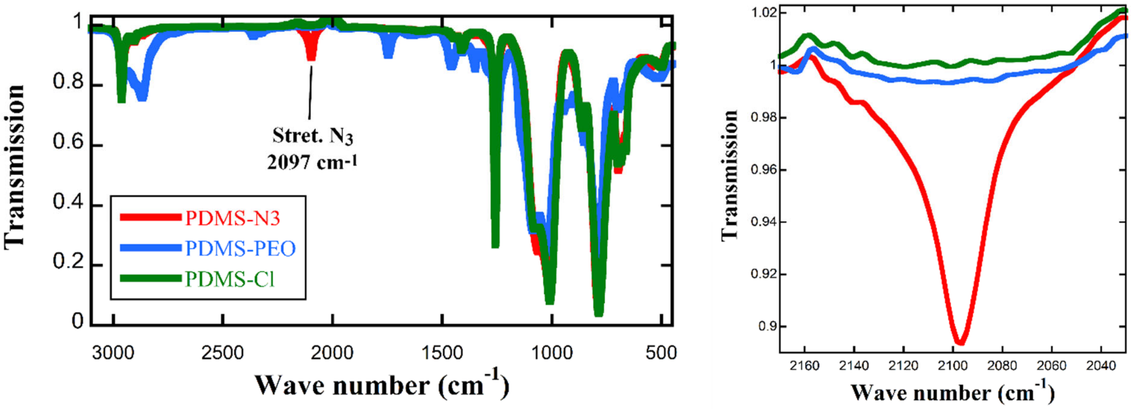

(a) **(b)**

Figure S3. Comparison of IR spectra of ω-chloro-PDMS$_{36}$, ω-azido-PDMS$_{36}$ and diblock copolymer PDMS$_{36}$-*b*-PEO$_{23}$. **B-** Zoom on characteristic peak of azide function at 2100 cm^{-1}.

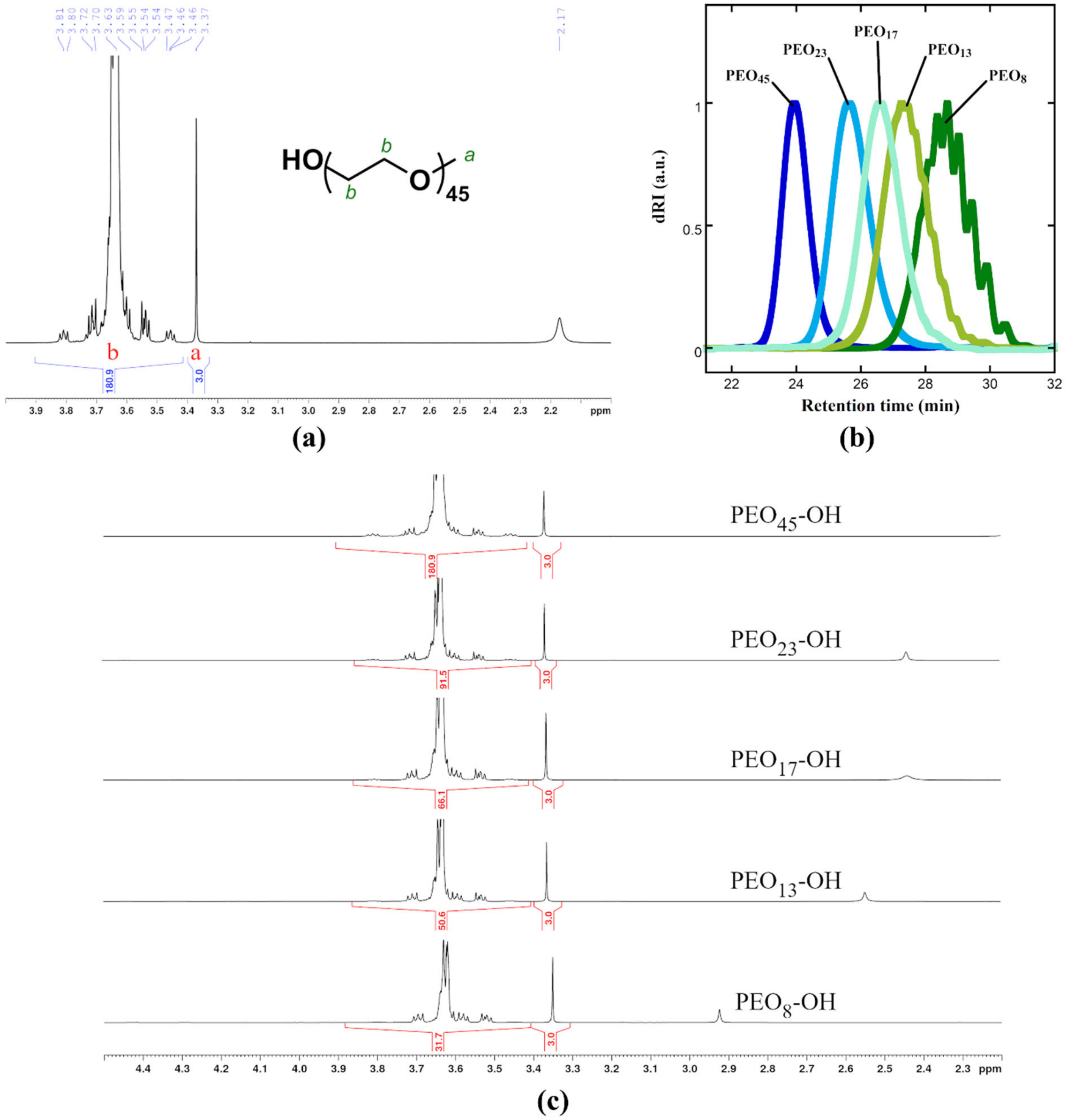

Figure S4. (a) and (c)^{1}H NMR spectra of the different commercial ω-hydroxy-PEO used. (b) SEC chromatograms of ω-hydroxy-PEO.

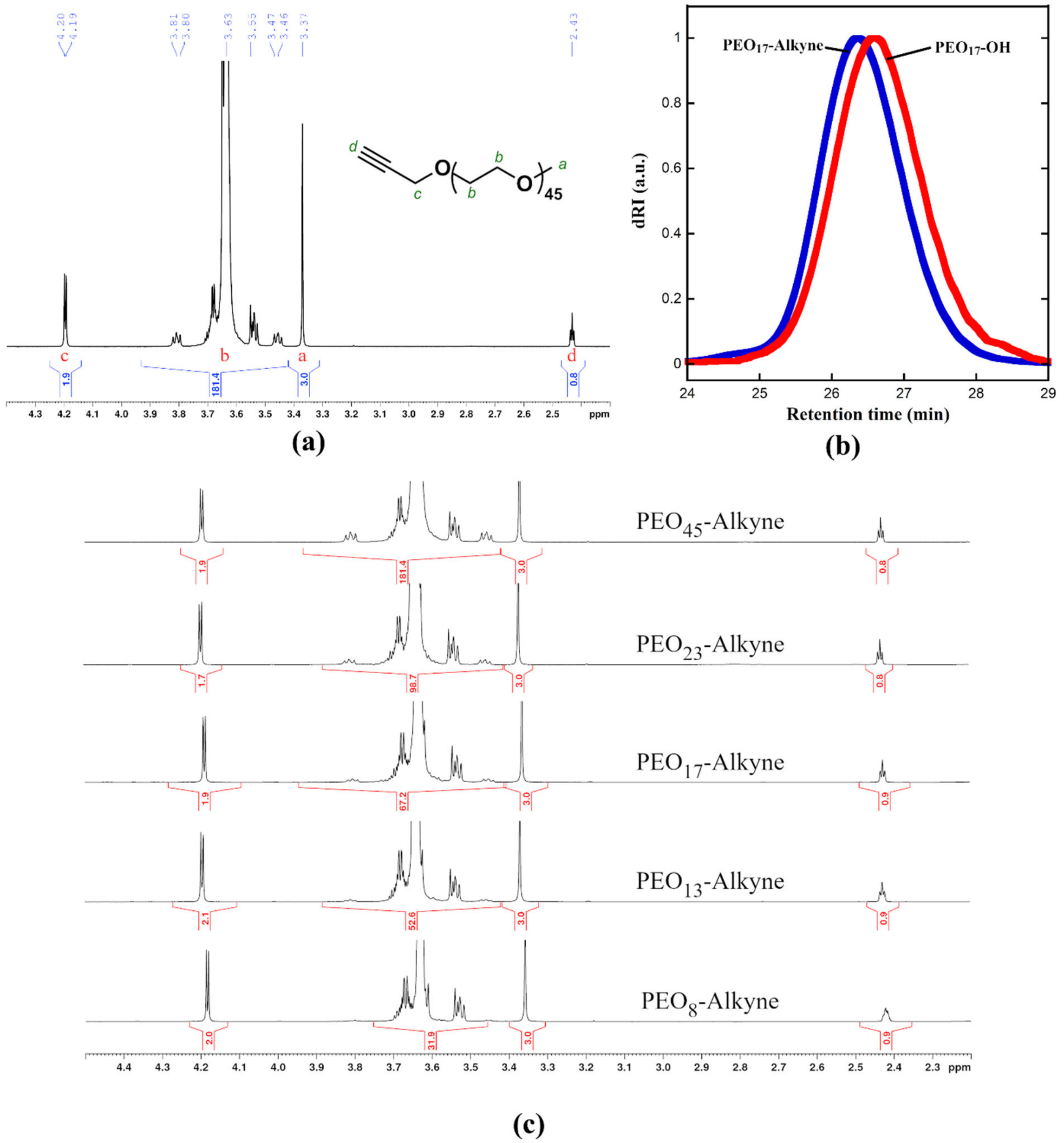

Figure S5. (a) and (c)^{1}H NMR spectra of the different ω-alkyne-PEO synthesized. (b) SEC of ω-alkyne-PEO_{17} and its precursor ω-hydroxy-PEO_{17}.

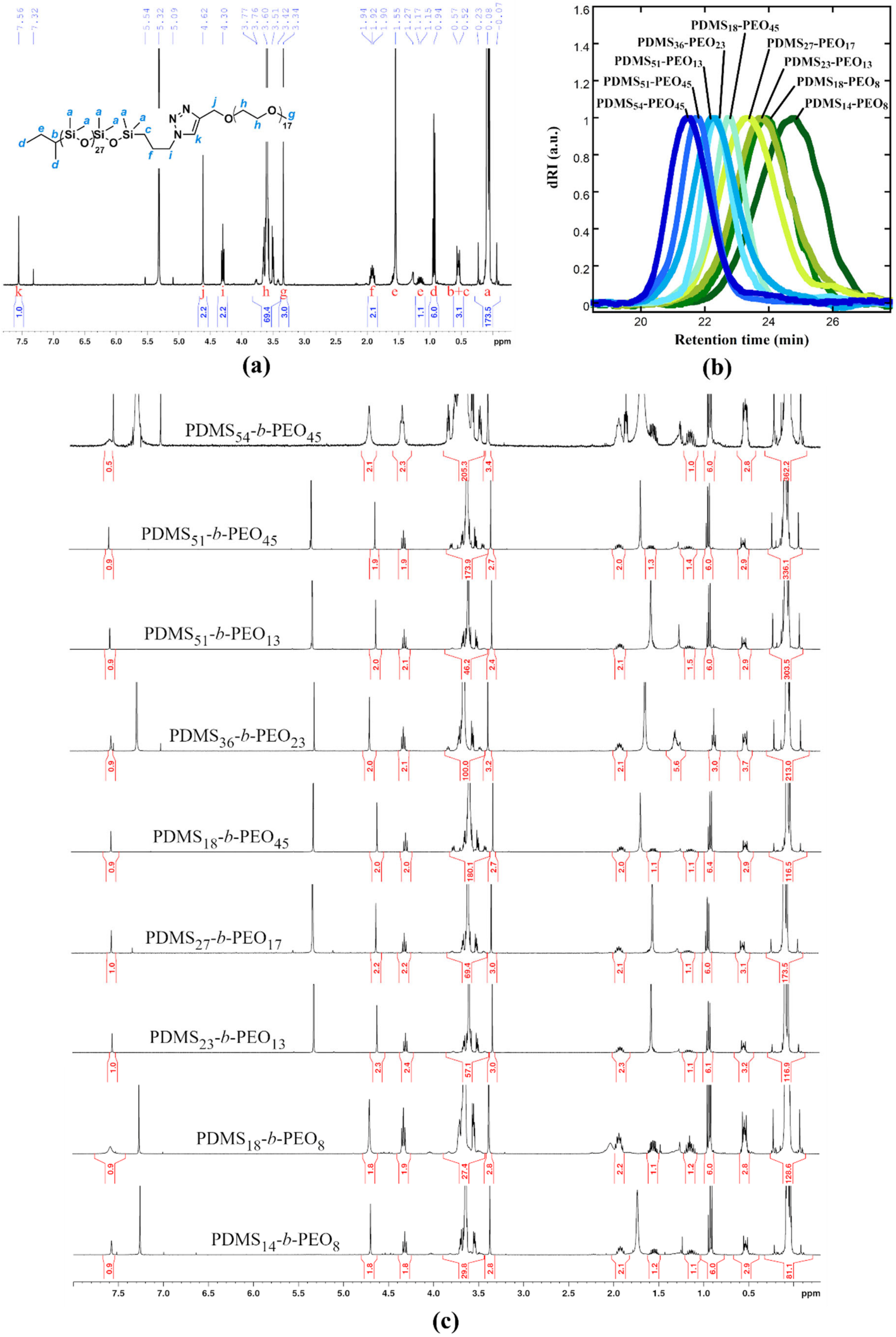

Figure S6. (a) and (c) ^{1}H NMR spectra of the different PDMS-*b*-PEO synthesized. (b)SEC chromatograms of PDMS-*b*-PEO diblock copolymers.

Table S1. Molecular characteristics of the different copolymers PDMS-b-PEO synthesised in this study.

	^{1}H NMR				SEC						
**Copolymers**	**$\bar{M}_n$ PDMS** (g.mol^{-1})	**$\bar{M}_n$ PEO** (g.mol^{-1})	**$\bar{M}_n$ copolymer** (g.mol^{-1})	**Hydrophylic weight fraction (%)**	**$\bar{M}_n$ PDMS** (g.mol^{-1})	**Đ PDMS**	**$\bar{M}_n$ PEO** (g.mol^{-1})	**Đ PEO**	**$\bar{M}_n$ copolymer** (g.mol^{-1})	**Đ copolymer**	**Hydrophylic weight fraction** (%)
**$PDMS_{54}$-*b*-PEO_{45}**	4000	2000	6200	33	4200	1,14	2300	1,03	7600	1,09	35
**$PDMS_{51}$-*b*-PEO_{45}**	3800	2000	6000	34	3300	1,08	2300	1,03	7400	1,08	41
**$PDMS_{51}$-*b*-PEO_{13}**	3800	600	4600	14	3300	1,08	600	1,11	5100	1,11	15
**$PDMS_{36}$-*b*-PEO_{23}**	2700	1000	4000	27	2700	1,09	1300	1,06	5000	1,04	33
**$PDMS_{27}$-*b*-PEO_{17}**	2000	700	2900	26	2000	1,18	900	1,04	3100	1,11	31
**$PDMS_{23}$-*b*-PEO_{13}**	1700	600	2500	26	1700	1,26	600	1,11	2500	1,15	26
**$PDMS_{18}$-*b*-PEO_{45}**	1300	2000	3500	61	1400	1,23	2300	1,03	4300	1,13	62
**$PDMS_{18}$-*b*-PEO_{8}**	1300	400	1900	24	1400	1,23	400	1,09	2700	1,08	22
**$PDMS_{14}$-*b*-PEO_{8}**	1000	400	1600	29	1000	1,12	400	1,09	1900	1,13	29

polymers MDPI

3. SANS characterization

Table S2. Fitting parameters of the SANS curves of block copolymers with vesicle form factor model.

Parameters	PDMS$_{14}$-*b*-PEO$_8$	PDMS$_{23}$-*b*-PEO$_{13}$	PDMS$_{27}$-*b*-PEO$_{17}$	PDMS$_{36}$-*b*-PEO$_{23}$
	Si$_{14}$EO$_8$	Si$_{23}$EO$_{13}$	Si$_{27}$EO$_{17}$	Si$_{36}$EO$_{23}$
Background (cm^{-1})	0.055	0.061	0.057	0.010
Scattering Length Density (x10^{-6} Å^{-2})		0.064		
SLD solvent (x10^{-6} Å^{-2})		6.360		
PDMS Volumic Fraction	0.0077	0.0099	0.0075	0.0067
Radius of Gyration (nm)	42	39	45	45
σ radius (log-normal distribution)		0.25		
Thickness (nm)	5.9	6.9	8.4	9.9
σ thickness (log-normal)	0.10	0.14	0.13	0.16

4. Micropipette experiments

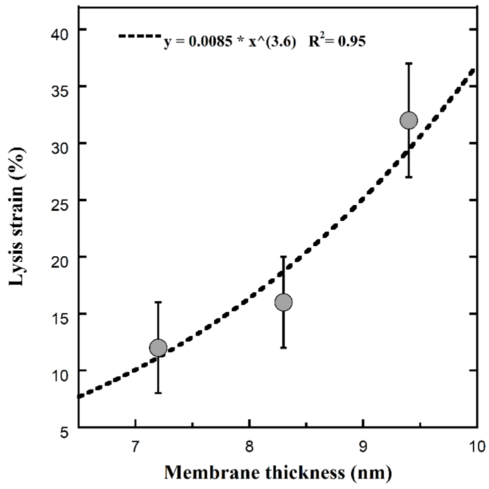

Figure S7. Lysis Strain versus membrane thickness for GUV obtained from PDMS-b-PEO diblock copolymers.

© 2019 by the authors. Submitted for possible open access publication under the terms and conditions of the Creative Commons Attribution (CC BY) license (http://creativecommons.org/licenses/by/4.0/).
